# Possible Improvement of the Sagittal Spinopelvic Alignment and Balance through “Locomotion Training” Exercises in Patients with “Locomotive Syndrome”: A Literature Review

**DOI:** 10.1155/2019/6496901

**Published:** 2019-04-08

**Authors:** Takashi Yurube, Masaaki Ito, Toru Takeoka, Nobuyoshi Watanabe, Hideyo Inaoka, Kenichiro Kakutani, Ryosuke Kuroda, Kotaro Nishida

**Affiliations:** ^1^Department of Orthopaedic Surgery, Kobe University Graduate School of Medicine, Kobe 650-0017, Japan; ^2^Department of Rehabilitation, Kyoto Kujo Hospital, Kyoto 601-8453, Japan; ^3^Department of Orthopaedic Surgery, Kyoto Kujo Hospital, Kyoto 601-8453, Japan

## Abstract

On the basis of rapid population aging, in 2007, the Japanese Orthopaedic Association (JOA) proposed a new disease concept “locomotive syndrome” as a degenerative condition of reduced mobility due to the impairment of the musculoskeletal system. Worsened locomotive components, which consist of bones, joints, and intervertebral discs, and muscles and nerves, can lead to symptoms such as pain, limited range of motion, malalignment, impaired balance, and difficulty in walking, ultimately resulting in the requirement of nursing care. “Locomotive syndrome” has gained increased interest in Japan but still not worldwide. Hence, in this brief review, we summarize an updated definition, assessment, and management of “locomotive syndrome”. The JOA recommends “locomotion training” exercise intervention to be effective in maintaining motor function that comprises two simple exercises—squatting and single-leg standing. However, the extent to which exercises affect “locomotive syndrome” is unknown. Here, we further report hypothesis-generating patient cases who presented the improved sagittal spinopelvic alignment in standing radiographs and postural stability in piezoelectric force-plate measurements through our 6-month “locomotion training” outpatient rehabilitation program. It is noteworthy that “locomotion training” facilitated these improvements despite the presence of specific disorders including thoracic kyphosis and symptomatic lumbar spinal canal stenosis. This raises the need for further investigations to clarify effects of “locomotion training” exercises on the spinal alignment, global balance, and quality of life in patients with “locomotive syndrome”.

## 1. Introduction

In 2016, the total population of Japan was 127 million, which was the 10th grade ranking country in the world [1]. However, the number of Japanese people in ages of 65 or more (recognized as the old age in Japan) was approximately 35 million, which was 27.3% of the entire population and the highest percentage of the aging rate in the world [2]. Hence, Japan has the most aged society.

In the aged Japanese population, a big issue is the gap between the increased actual life span and the healthy life expectancy. In 2013, this gap was approximately 9 years in Japanese men and 12 years in Japanese women [3]. Furthermore, the primary cause of the requirement of nursing care in elderly Japanese persons was musculoskeletal disorders (24.6% in 2016), which was higher than cerebrovascular disease and dementia [3]. Actually in persons with any subjective symptoms, low back pain, stiff neck and shoulder, and peripheral joint pain occupied the 1st, 2nd, and 5th and 1st, 2nd, and 3rd most frequent complaints in Japanese men and women, respectively [3]. Therefore, the maintenance of healthy musculoskeletal functions is an urgent national concern in Japan.

On the basis of this accelerated population aging, in 2007, the Japanese Orthopaedic Association (JOA) determined a new disease concept “locomotive syndrome” as a condition being restricted in the ability to walk and have a normal life owing to a degenerative dysfunction in one or more of the parts of the musculoskeletal system [4, 5]. The musculoskeletal system which can cause “locomotive syndrome” consists of three major components: (1) bones, (2) joints and intervertebral discs, and (3) muscles and nerves. The age-related impairment of these organs causes (1) osteoporosis and related fragile fractures, (2) osteoarthritis and spondylosis, and (3) sarcopenia and neural disorders. These diseases can lead to symptoms of pain, limited range of joint motion, malalignment, imbalance, and then difficulty in standing-up and walking, finally resulting in reduced activities of daily living (ADL), lower quality of life (QOL), and required nursing care. “Locomotive syndrome” is the basis of musculoskeletal healthcare problems in the present Japanese society.

Degenerative changes in the musculoskeletal system appear before middle ages. The intervertebral disc is one of the earliest organs to develop degeneration in the body [6]. The first unequivocal findings of degeneration in the lumbar discs are seen in the age group 11–16 years [7]. Then, ~40% of people in ages under 30 years and 90% of those in ages over 55 years present lumbar disc degeneration [8]. Furthermore, a Japanese cohort study found that the estimated number of patients with radiographic knee osteoarthritis, lumbar spondylosis, lumbar osteoporosis, and femoral neck osteoporosis were 25, 38, 6.4, and 11 million of 128 million (Japan's 2005 population) [9]. The prevalence of these disorders is quite high, requiring heavy socioeconomic burden. Therefore, health promotion to prevent “locomotive syndrome” is essential.

## 2. Assessment of “Locomotive Syndrome”

Early diagnosis and recognition are important. To identify people at risk of “locomotive syndrome” and also raise the public interest in the importance of “locomotive syndrome”, JOA developed “loco-check” self-completed questionnaire [10]. Although self-assessment by using “loco-check” is not a mandatory step toward the diagnosis with “locomotive syndrome”, “loco-check” is a useful self-assessment tool to detect early stages of “locomotive syndrome” and initiate preventive measures. In fact, statistical correlation between the number of positive “loco-check” items and the incidence of falling in the previous year was found [11].

Many functional assessments, such as the hand-grip strength, one-leg standing time, and 6 m walking time, were proposed to evaluate “locomotive syndrome” [12, 13]. These measurements are useful; however, further validations are required. Currently, the stand-up test, two-step test, and 25-question geriatric locomotive function scale (GLFS-25) have been officially introduced for the diagnosis with “locomotive syndrome” [10, 14]. In these assessments, two risk levels of “locomotive syndrome” have been categorized to assess the severity. To determine “locomotive syndrome risk level”, all these three tests (stand-up test, two-step test, and GLFS-25) have to be completed. Recent literature has often recognized “locomotive syndrome risk level 2” as patients who have positive “locomotive syndrome” [15].

### 2.1. Loco-Check

“Loco-check” consists of the following seven statements regarding daily activities: (1) you cannot put your sock on standing on one leg; (2) you often trip up or slip around the house; (3) you need to hold on to the handrail when climbing the stairs; (4) you have difficulty in doing moderately heavy housework; (5) you have difficulty in carrying home 2 kg of shopping; (6) you cannot walk for a quarter of an hour nonstop; and (7) you cannot make it across the road before the light turns red. Persons who meet one or more statements are suspected of having “locomotive syndrome” [10, 11].

### 2.2. Stand-Up Test

Subjects are asked to stand from stools of varying heights (10, 20, 30, and 40 cm) with a single leg and both legs [16]. First, the trial using the 40 cm stool is performed with both legs. When completed, the 40 cm stool is tried with a single leg, followed by the 30 cm, 20 cm, and then 10 cm stools. When the 40 cm stool with a single leg is not completed in both right and left legs, the 30 cm, 20 cm, and then 10 cm stools are tried with both legs. Consequently, subjects who fail to stand from the 40 cm stool with a single leg in either of bilateral legs are regarded as “locomotive syndrome risk level 1”. Then, subjects who fail to stand from the 20 cm stool with both legs are regarded as “locomotive syndrome risk level 2” [10, 14].

### 2.3. Two-Step Test

Subjects begin the two-step test in an upright posture and move forward for a maximum of two strides without losing his or her balance [16]. The better result after two trials is recorded. The two-slide distance is subsequently standardized according to the subjects' height. Consequently, subjects with the value less than 1.3 and 1.1 are regarded as “locomotive syndrome risk level 1” and “locomotive syndrome risk level 2”, respectively [10, 14].

### 2.4. Twenty-Five-Question Geriatric Locomotive Function Scale

The GLFS-25 is a self-reported tool to assess difficulty and disability in daily activities related to locomotive organs [17]. This questionnaire consists of total 25 questions (4 questions regarding pain, 16 questions regarding ADL, 3 questions regarding social functions, and 2 questions regarding mental health status) that refer to experiences in the preceding month. The answer of each question is rated on a scale of 0–4 points, indicating higher scores as the presence of symptoms and medical conditions resulting from a greater severity of “locomotive syndrome” (total minimum 0–maximum 100). After careful statistical analysis and evaluation [17], patients with 7 or more points of the GLFS-25 score and those with 16 or more points are regarded as “locomotive syndrome risk level 1” and “locomotive syndrome risk level 2”, respectively [10, 14].

## 3. Prevalence of “Locomotive Syndrome”

Nationwide surveys reported the prevalence of “locomotive syndrome (risk level 2 by the GLFS-25 score ≥16)” in Japanese people in ages of 40 years or more as 10.2% (men, 7.9%; women, 12.3%) in 2010 [18] and 11.9% (men, 10.8%; women, 12.9%) in 2014 [19]. The age-specific mean values for GLFS-25 were 5.8, 6.0, 5.9, and 8.8 of 100 points in the 40s, 50s, 60s, and 70s, respectively [18]. The mean value for GLFS-25 was higher in the 70s than in the other age groups and in women than in men [18]. Then, the longitudinal 7-year follow-up survey of the Research on Osteoarthritis/Osteoporosis Against Disability study found the estimated 2013 prevalence in Japan of the indices in “locomotive syndrome risk level 2” including the two-step test score <1.1, difficulty in standing from a 20 cm height using both legs in the stand-up test, and GLFS-25 score ≥16 as 21.1% (men, 20.1%; women, 21.6%), 7.9% (men, 4.9%; women, 9.4% [statistically higher than men]), and 10.6% (men, 9.0%; women, 11.4%), respectively [14]. After the calculation, the estimated Japan's 2013 prevalence of “locomotive syndrome (risk level 2)” was 25.1% (men, 22.7%; women, 26.3%) [20]. This prevalence was significantly higher with aging, although there was no statistical sex difference [20]. Hence, the prevalence of “locomotive syndrome” primarily increases by aging, especially in patients in ages of 70 years or more, which is more likely to develop in women.

## 4. Intervention in “Locomotive Syndrome”

“Locomotive syndrome risk level 1” indicates that the impairment of locomotive functions has already begun. As muscle strength and posture balance may be deteriorating, JOA recommends to perform daily physical exercises such as “locomotion training” in subjects with “locomotive syndrome risk level 1”. In addition, it is recommended to take care to eat a balanced diet with plenty of protein and calcium [10, 21].

“Locomotive syndrome risk level 2” indicates that the impairment of locomotive functions has already progressed. Subjects with “locomotive syndrome risk level 2” are at high risk of having the difficulty in keeping an independent life style. As the subjects may have locomotive organ disorders, JOA recommends to continue exercise training and also medical consultation to orthopaedic clinics [10].

## 5. “Locomotion Training”

Many studies have reported effectiveness of physical intervention in preventing the loss of mobility, balance, and gait in the geriatric population [22–32], while exercises are generally effective in subjects with mild to moderate disability [24, 33] but not so much in subjects with severe disability [26]. Thus, early detection of “locomotive syndrome” is desirable.

Physical intervention is based on the principles of exercise [34]. First, it is known that the particular body components and skills, which are involved in a given exercise, will demonstrate the improvement (principle of specificity). Second, a high load is required for any functional improvement (principle of overload). Third, it is important to gradually increase the exercise load (principle of progression) with consideration for safety since the majority of people in middle to old ages have chronic degeneration of lumbar spine discs and lower limb cartilages [9].

Therefore, JOA recommends “locomotion training” to improve and sustain standing and gait functions in middle-aged and old-aged subjects [5, 10]. The basic protocol of “locomotion training” just consists of two simple exercises directly related to standing—squatting and single-leg standing [35]. Then, in patients who get used to the basic “locomotion training” exercises, other exercises such as heel raises and front lunges are recommended to be added [10]. Walking is generally recommended [36–38]. However, persons with the GLFS-25 score ≥16 are expected to have trouble in walking and going out [17]. Three “locomotive syndrome risk level” indices—the stand-up test, two-step test, and GLFS-25—all predict immobility significantly and independently, and the accumulation of these indices indicates substantial increases in the risk of immobility [14]. Therefore, overload is unfavourable. The JOA recommends just additional 10-minute mild physical activities which can support the prevention of “locomotive syndrome”, e.g., bicycling or walking to work, taking the stairs instead of the elevator or escalator, cleaning, and laundering with zest in addition to stretching when you have a moment, doing “locomotion training” or stretching while watching TV, taking a walk during breaks at the office, walking to a supermarket further away than usual for your shopping, using your local park or sports center, taking part in community sports events, going out to have fun with family or friends on days off, and walking briskly with long strides [5, 10].

### 5.1. Single-Leg Standing Exercise

The single-leg standing exercise is designed to improve posture balance. This exercise can be done alone [39] or combined with other muscle power training (like chair-rising training) [40]. This test has been demonstrated to be effective in preventing falls [39, 40]. Subjects are instructed to stand on one leg with their eyes open and adjacent to a stable chair or desk for arm support to prevent from falling. Performing one minute for each leg, three times a day (morning, noon, and evening), every day is advised.

### 5.2. Squatting

The squatting exercise is designed to strengthen leg muscles. Previous studies have demonstrated effectiveness of squatting in reducing bone loss and improving muscle strength and balance in the lower extremities [41, 42]. Subjects slowly move the torso down from the standing position as is done during stand–sit movement. In addition, subjects are instructed to maintain the position of the patella over the toes to prevent overload on the knee. The knee flexion angle should not exceed 90°. Performing slow squats five to six times as one set, three times a day, every day is recommended.

## 6. Effect of “Locomotion Training”

Only a few English reports describing the improvement of physical function tests (visual analog scale scores of low back pain, single-leg standing time, and 6 m walking time) by “locomotion training” intervention have been published [43]. A prior study demonstrated that “locomotion training” monitored by using serial telephone contacts improved physical function test scores and seven of eight SF-8 subscales [44]. Low back pain decreased in 12.6% of the subjects while it increased only in 2.3%. Knee pain decreased in 17.2% of the participants while it increased only in 1.1%. Hence, evidence is accumulating regarding subjective improvements of physical functions, ADL, and QOL through “locomotion training” exercises. However, multidisciplinary studies regarding objective improvements, e.g., radiography, magnetic resonance imaging, electromyography, and bone densitometry, still need to be conducted. These measurements provide useful information to elucidate effects of “locomotion training” on the spinal and upper and lower extremities' alignment, muscle size and quality, and respective disease severity including osteoarthritis, spinal canal stenosis, sarcopenia, and osteoporosis, as the progress of degenerative disorders is not always symptomatic.

## 7. Future Directions

In age-related changes in the musculoskeletal system, adult deformity of the spine gains increased attention [45]. Particularly, the loss of lumbar lordosis was associated with increased pain and disability and lower QOL scores [46]. The stronger the back extensors, the smaller the thoracic kyphosis and the larger the lumbar lordosis and sacral inclination [47]. In prior reports studying “Pilates”-based exercises, significant improvement in the sagittal alignment of the head was observed after 6-month exercises; however, this was not the report of radiographic measurements [48].

Based on literature evidence, a prospective cohort study was designed and conducted to assess effects of “locomotion training”-based exercises on the sagittal alignment of the spinopelvic axis in standing radiographs and postural balance in piezoelectric force-plate measurements. All experimental procedures were performed under the approval and guidance of the Institutional Review Board at Kyoto Kujo Hospital (Kyoto, Japan). Written informed consent was obtained from each patient in accordance with the principles of the Declaration of Helsinki and the laws and regulations of Japan. In this paper, we would like to show representative patient cases with varying pathologies who underwent our 6-month “locomotion training” outpatient rehabilitation program.

**Figure 1:**
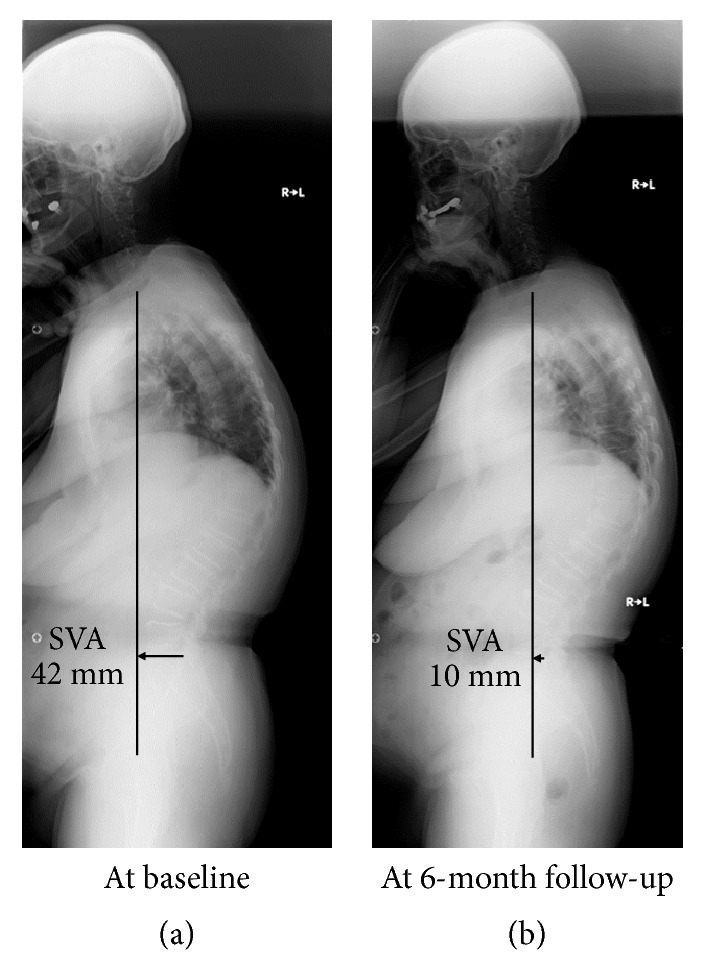
A 70-year-old woman with “locomotive syndrome risk level 2” from lumbar spondylosis. Standing whole-spine radiographs showed the sagittal vertical axis (SVA) of 42 mm with thoracic kyphosis (a). After our 6-month “locomotion training” outpatient rehabilitation program, she showed the improvement to “locomotive syndrome risk level 1” with the improved SVA up to 10 mm (<40 mm, normal range) and decreased thoracic kyphosis in radiographs (b).

A 70-year-old woman experienced low back pain lasting over 2 months and then visited our orthopaedic clinic. Her diagnosis was lumbar spondylosis. At baseline, she showed standing-up from a 40 cm height on both legs at her best on the stand-up test (risk level 2), 1.05 on the two-step test (risk level 2), and 14 points on the GLFS-25 score (risk level 1), resulting in the categorization into “locomotive syndrome risk level 2”. Then, in standing whole-spine radiographs with the clavicle position, baseline sagittal vertical axis (SVA) was 42 mm, indicating mild sagittal deformity [49]. The lumbar lordosis between L1 and S1 (LL) and pelvic incidence (PI) were 38° and 37°, respectively, causing no mismatch of the PI−LL [49]. The pelvic tilt and sacral slope were 16° and 21°, respectively. Furthermore, C2–C7 angle, T1 slope, and thoracic kyphosis between T5 and T12 (TK) were 16°, 40°, and 51°, respectively, thus suggesting that her sagittal deformity primarily resulted from thoracic kyphosis despite no vertebral fractures (Figure 1(a)). She attended our “locomotion training” outpatient rehabilitation program for 20 minutes, once a week, 6 months to confirm the achievement of exercises and add stretching. At endpoint, she improved “locomotive syndrome” scores to complete standing-up from a 20 cm height on both legs on the stand-up test (risk level 1), 1.2 on the two-step test (risk level 1), and 9 points on the GLFS-25 score (risk level 1), corresponding to “risk level 1”. The SVA improved up to 10 mm (<40 mm, normal range) [49] with the LL of 42°. Notably, C2–C7 angle, T1 slope, and TK improved to 4°, 29°, and 47°, respectively (Figure 1(b)). The Oswestry Disability Index (ODI) to assess low back pain and related QOL improved from 10 (22.2%, moderate disability) to 7 (15.6%, minimal disability). In piezoelectric force-plate measurements, baseline and endpoint areas of the pressure center, speed, and distance were 7.90 cm^2^ and 6.79 cm^2^, 3.18 cm/s and 2.00 cm/s, and 190.60 cm and 119.75 cm, respectively, all of which were improved.

**Figure 2:**
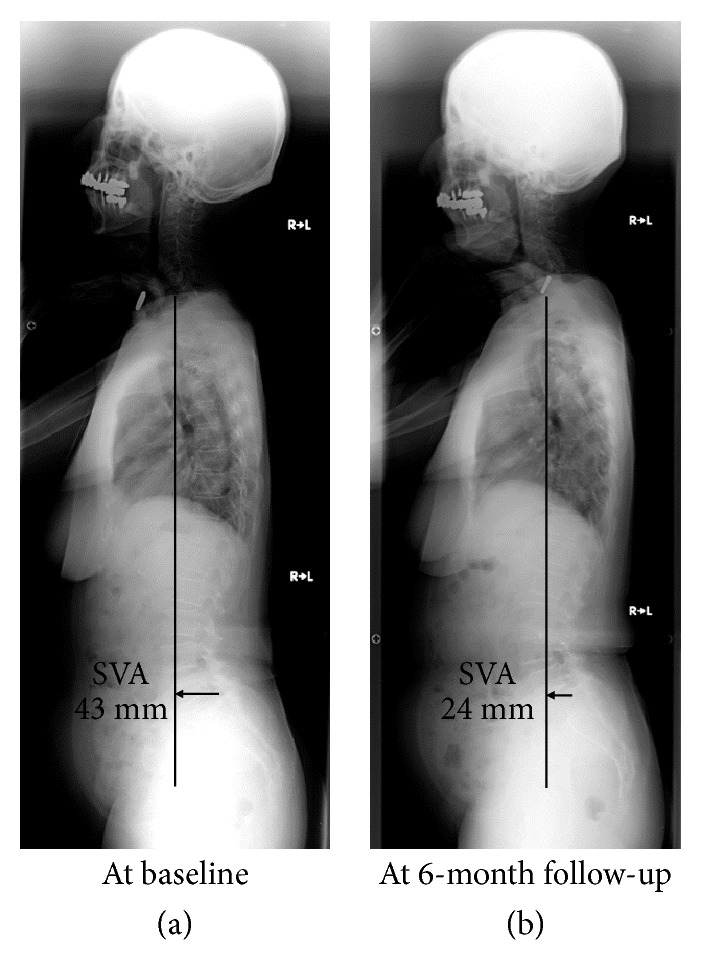
A 72-year-old woman with “locomotive syndrome risk level 2” from lumbar spinal canal stenosis. Standing whole-spine radiographs showed the sagittal vertical axis (SVA) of 43 mm with decreased thoracic kyphosis (a). After our 6-month “locomotion training” outpatient rehabilitation program, she showed the improvement to “locomotive syndrome risk level 1” with the improved SVA up to 24 mm (<40 mm, normal range) and increased thoracic kyphosis in radiographs (b).

A 72-year-old woman experienced low back and bilateral buttock to leg pain worsened only during housework and walking. Her symptoms were relieved after sitting. She visited our orthopaedic clinic, and magnetic resonance imaging analysis found lumbar spinal canal stenosis at L4–L5. Baseline and endpoint “locomotive syndrome” scores were 30 cm on both legs (risk level 2) and 20 cm on both legs (risk level 1) on the stand-up test, 1.1 (risk level 1) and 1.3 (risk level not applicable) on the two-step test, and 8 points (risk level 1) and 5 points (risk level not applicable) on the GLFS-25, respectively (overall risk level 2 → 1). Baseline SVA, LL, and PI−LL were 43 mm, 49°, and +4°, respectively (Figure 2(a)). Then, endpoint SVA, LL, and PI−LL were 24 mm, 48°, and +5°, respectively (Figure 2(b)). Thus, SVA improved to the normal range after “locomotion training” exercises. Also in this case, TK decreased to 26° at baseline but increased up to 38° at endpoint. Baseline and endpoint ODI were 8 (17.8%, minimal disability) and 6 (13.3%, minimal disability), no remarkable deterioration of which was observed. However, she recognized substantial improvement of intermittent claudication. Force-plate examination showed the improvement in part of the area, speed, and distance: 4.47 cm^2^ and 2.76 cm^2^, 1.53 cm/s and 1.54 cm/s, and 91.69 cm and 92.54 cm at baseline and endpoint, respectively.

The first case's woman had the maintained lumbar lordosis and pelvic parameters but increased thoracic kyphosis. This patient case may be a good candidate of “locomotion training” rehabilitation program. Strengthened leg, hip, and back muscles can facilitate the improvement of the sagittal alignment of the spine. Then, the second case's woman suffered from intermittent claudication. The applied “locomotion training” rehabilitation program improved the sagittal alignment and reduced complaints of intermittent claudication. While the first case decreased the patient's own thoracic kyphosis, the second case increased the thoracic kyphosis. Literature evidence reported a normal range of TK (T5–T12) as 34°  ± 11° [50]. Improved power and flexibility of the lower limb, hip, and back muscles might contribute to the normalization of the sagittal alignment of the thoracic spine. It is noteworthy that “locomotion training” facilitated these improvements despite the presence of specific disorders including thoracic kyphosis and symptomatic lumbar spinal canal stenosis.

## 8. Conclusions

The cases shown raised the need for further prospective cohort studies to clarify multidisciplinary effects of “locomotion training” exercises on the spinal alignment, global balance, and quality of life in patients with “locomotive syndrome”. Understanding of “locomotive syndrome” is essential for the future of aging care, which is an important health issue not only in Japan but also in the international society.

## Data Availability

All the data used to support the findings of this study are included within the article.
